# An Adipokinetic Hormone Acts as a Volume Regulator in the Intertidal Gastropod Mollusk, *Aplysia californica*

**DOI:** 10.3389/fendo.2018.00493

**Published:** 2018-08-24

**Authors:** Anthony W. Martillotti, Pei-San Tsai

**Affiliations:** Department of Integrative Physiology, University of Colorado, Boulder, CO, United States

**Keywords:** adipokinetic hormone, osmoregulation, volume regulation, gonadotropin-releasing hormone superfamily, neuropeptide, neurohormone, evolution

## Abstract

Adipokinetic hormone (AKH) is a multifunctional neuropeptide in the gonadotropin-releasing hormone superfamily. In insects, AKH acts to mobilize energy stores during times of high energetic demand, but has been shown to have other effects. In lophotrochozoans, the presence and function of AKH are less characterized. We have previously identified an AKH in an intertidal gastropod mollusk, the California sea hare (*Aplysia californica*), and named it ac-AKH. Our previous data showed ac-AKH induced an acute weight loss, suggesting a role in volume regulation. The overarching goals of this study were to test the role of ac-AKH as a volume regulator and examine the mechanism by which ac-AKH induced the acute weight loss. Our results showed that ac-AKH reduced body mass, in part, through the reduction of hemolymph volume without altering hemolymph osmolality or specific osmolytes. The effect of ac-AKH on volume loss was accentuated under a hyposaline condition. We further showed that *ac-akh* expression was inhibited during a hyposaline challenge, and that the administration of ac-AKH partially reversed the increase in body mass, but not hemolymph osmolality change, caused by the hyposaline challenge. These data collectively show that ac-AKH is a proximate regulator controlling the fluid volume, but not osmolality, in *A. californica*. Importantly, our results highlight the functional divergence of this structurally conserved neuropeptide in the molluscan lineage.

## Introduction

Adipokinetic hormone (AKH) is a small neuropeptide hormone and a member of the gonadotropin-releasing hormone superfamily. The presence and function of AKH are best characterized in the arthropods (1, 2). In insects, it is produced in the corpora cardiaca and chiefly released during times of high energetic demand to mobilize energy stores, including lipids, carbohydrates, and proteins (3–6). However, its function is not restricted to energetic balance. AKH also has a role in the stress response (7), and red pigment-concentrating hormone (RPCH), a crustacean homolog of insect AKH and a member of the AKH family (8, 9), mobilizes pigments and has a stimulatory effect on female reproduction (10). Therefore, AKH is considered a multifunctional neuropeptide capable of functions beyond the regulation of energetic balance.

We have previously characterized the structure and function of an AKH in a gastropod mollusk, *Aplysia californica* (11). The *A. californica* AKH (ac-AKH) was the first lophotrochozoan AKH to be characterized and showed several novel functions. While the chronic administration of ac-AKH resulted in increased hemolymph glucose, acute administration resulted in enhanced excretion of feces, inhibition of feeding, and a rapid loss of over 20% body mass within the first 6 h (11). The magnitude and rapid nature of the body mass loss induced by ac-AKH suggested the mechanism underlying this effect was water loss.

Osmoregulation and hemolymph volume regulation in gastropod mollusks are two poorly understood physiological processes. *A. californica* are intertidal animals that need to withstand substantial fluctuations in seawater salinity (12, 13), thus osmoregulation and volume regulation are critically required for their survival. Literature on this subject using the genus *Aplysia* is limited and typically focused on the electrophysiology of the burst neuron R15, a neuron in the abdominal ganglion thought to be involved in osmoregulation (14–19). One of the few studies on osmoregulation and volume regulation at the level of the organism (20) showed that *A. californica* transferred to a 90% artificial seawater (ASW) environment initially experienced reduced hemolymph osmolality and weight gain but later returned to their original osmolality and weight in 24 h, suggesting effective mechanisms of osmoregulation and hemolymph volume regulation within a salinity range. Based on our previous results that ac-AKH induced a rapid weight loss in *A. californica*, possibly through water loss (11), we hypothesized that ac-AKH may be involved in the processes of osmoregulation and volume regulation that allow the animals to cope with declining environmental salinity.

The objectives of this study were threefold. First, we determined if a single injection of ac-AKH altered the body mass and hemolymph osmolality of *A. californica* kept under different salinity conditions, and if the change in body mass was due, in part, to the change in hemolymph volume. Secondly, we determined if the transfer of *A. californica* from a normosaline to hyposaline condition altered the expression of *ac-akh*. Lastly, we treated the transferred animals with ac-AKH to determine if ac-AKH could reverse the weight gain induced by a normosaline to hyposaline transfer. Our results show, for the first time, the ability of an AKH to reduce hemolymph volume and reverse hyposaline-induced weight gain without a concurrent change in hemolymph osmolality. Further, the expression of *ac-AKH* was significantly reduced during a normosaline to hyposaline transfer when the animals gained water. Collectively, our results suggest ac-AKH has a physiological role in actively reducing body fluid content, and the reduced presence of this peptide may be needed for fluid gain. These results further highlight the functional diversity of the AKH peptide family and uncover a possible role of AKH in the molluscan lineage.

## Materials and methods

### Animals

Adult *A. californica* (between 92.2 and 216.6 g) were purchased from Alacrity Marine Biological Services (Redondo Beach, CA) or the National Resource for Aplysia (Miami, FL). Animals were housed individually in perforated floating cages in a recirculating system containing 300 gallons of Instant Ocean (Spectrum Brands, Blacksburg, VA) held at 15–18°C. All animals were acclimated to the experimental salinity for 48–72 h before use. Unless specified, all animals were anesthetized with 1/3 body mass of isotonic MgCl_2_ immediately prior to sacrifice. For some experiments, animals were bled by quickly drawing hemolymph from the posterior foot using a 22-gauge needle at specified time points. Although institutional approval was not required for the use of *A. californica*, every effort was made to adhere to standards of humane treatment for the care and use of these animals.

### Experiment 1: effects of ac-AKH injection on body mass and hemolymph osmolality

To assess the impact of ac-AKH on body mass and hemolymph osmolality under different salinity conditions, animals were acclimated to either a normosaline (997 mOsm/L) or a hyposaline (779 mOsm/L) environment. An osmolality of 997 mOsm/L is similar to oceanic osmolality (21), and 779 mOsm represents a 20% reduction. A baseline body mass was taken prior to a single injection of either 500 μL artificial seawater (ASW; 395 mM NaCl, 10 mM KCl, 10 mM CaCl_2_, 50 mM MgCl_2_, 28 mM Na_2_SO_4_, and 30 mM HEPES) or 10 μg ac-AKH in 500 μL ASW. The dose of injected ac-AKH was estimated to produce ~35-50 nM circulating peptide in the absence of protease degradation (11), a range consistent with the *in vivo* and *in vitro* treatments of many GnRH superfamily peptides (22–25). Animals were blotted and additionally weighed at 1, 2, 4, 6, and 24 h after the injection. To assess the impact of ac-AKH on hemolymph osmolality, 500 μL of hemolymph was collected at 0, 6, and 24 h after the injection. Hemolymph was cleared by centrifugation at 3000 rpm and the supernatant stored at −20°C until osmolality measurement using a freezing-point depression osmometer. A fraction of hemolymph samples collected at 0 and 6 h were sent to Comparative Clinical Pathology Services, LLC (Columbia, MO) where ion-specific electrodes and commercial enzyme kits were used to assess concentrations of ions (Na^+^, K^+^, Cl^−^, and Ca^2+^), glucose, proteins, and triglycerides with a Beckman-Coulter AU680 automated chemistry analyzer (Brea, CA).

### Experiment 2: the effects of ac-AKH on hemolymph volume

To determine if weight loss induced by ac-AKH was due to a reduction in the hemolymph volume, terminal hemolymph volume was measured in animals injected with 500 μL ASW vehicle or 10 μg ac-AKH in 500 μL vehicle. For this experiment only, animals were anesthetized by a 30-min period of cold narcosis at 6 h after injection. Animals were firmly wrapped with a protective diaper to absorb contaminating mucus and ink and exsanguinated through a small opening made at the base of the foot by gentle manual squeezing. The entire hemolymph volume was collected into a beaker for future volume measurements. In our hands, animals never excrete mucus or ink after 30 min of cold narcosis. The standard procedure was followed to ensure all animals were bled until no more hemolymph could be collected after 90 s of repeated squeezing. Although the investigators were not blind to the treatment conditions, they were blind to the volume of the hemolymph collected until the volume measurements were performed. Animals were weighed immediately prior to the injection and exsanguination.

### Experiment 3: localization of ac-AKH in peripheral and central tissues

To examine the pattern of ac-AKH expression, tissues were collected from control animals previously injected with 500 μL ASW (from Experiment 1). The central nervous system (CNS), heart, kidney, gill, esophagus, crop, and intestine were harvested, snap-frozen on dry ice, and stored at −70°C until RNA isolation. Total RNA was isolated using TRIzol reagent (Ambion, Austin, TX) according to the manufacturer's instructions. cDNA synthesis was performed from 1 μg total RNA using the QuantiTect Reverse Transcription Kit (Qiagen, Germantown, MD) according to the manufacturer's instructions.

For quantitative reverse-transcription PCR (qPCR), *A. californica* actin (*ac-actin*) was used as a reference gene to normalize the target gene expression. qPCR primers for *ac-akh* were designed based on the previously published sequence (11). qPCR of *ac-akh* was carried out using FastStart Universal SYBR Green mix (Roche, Indianapolis, IN) and calculated using the 2^−ΔΔ*CT*^ method (26). Each reaction contained 9 μL SYBR Green mix, 9 μL ultrapure water, 0.5 μM gene-specific primers, and 2 μL (for *ac-actin* and *ac-akh*) of cDNA. Forward and reverse primers were 5′-CACACTGTCCCCATCTACGA and 5′-CCAGCGAGATCCAATCTCAT for *ac-actin*, and 5′-TTAATACAGCGAACCGCAAA, and 5′-TCACAGTTCTGGGCAGGTATT for *ac-akh*.

### Experiment 4: alteration of *ac-AKH* expression during normosaline to hyposaline transfer

*A. californica* were transferred from a normosaline (1003 mOsm/L) to a hyposaline (757 mOsm/L) condition to determine the effects of salinity change on body mass and the expression of *ac-akh*. Animals were weighed at 1, 2, 4, and 6 h after the transfer and sacrificed at 6 h to harvest the hemolymph for osmolality measurement, and CNS, heart, kidney, and intestine for *ac-akh* transcript quantification by qPCR as described above for Experiment 3.

### Experiment 5: the effects of ac-AKH on hyposaline transfer

Since ac-AKH significantly reduced body mass in Experiment 1 under both salinity conditions, we assessed if ac-AKH injection could reverse the weight gain induced by transfer to a hyposaline condition. Animals were subject to one of the following three treatments: (1) injected with 500 μL ASW vehicle and retained in a normosaline condition (984 mOsm/L), (2) injected with 500 μL ASW vehicle and transferred to a hyposaline condition (744 mOsm/L), or (3) injected with 10 μg ac-AKH in 500 μL vehicle and transferred to a hyposaline condition. Animals were weighed immediately before each hemolymph draw at 0, 0.5, 1, 2, 4, 6, and 8 h.

### Statistical analysis

Repeated measurements of changes in body mass and hemolymph parameters were analyzed using two-way repeated measures ANOVA followed by Bonferroni's *post-hoc* test to determine significant difference between treatment groups at each time point. For Experiment 2, statistical analysis was not performed due to the descriptive nature of the expression profiling. Difference between two independent groups was otherwise analyzed by Student's *t-*test with Welch's correction.

## Results

### Experiment 1: effects of ac-AKH injection on body mass and hemolymph osmolality

**Figure 1:**
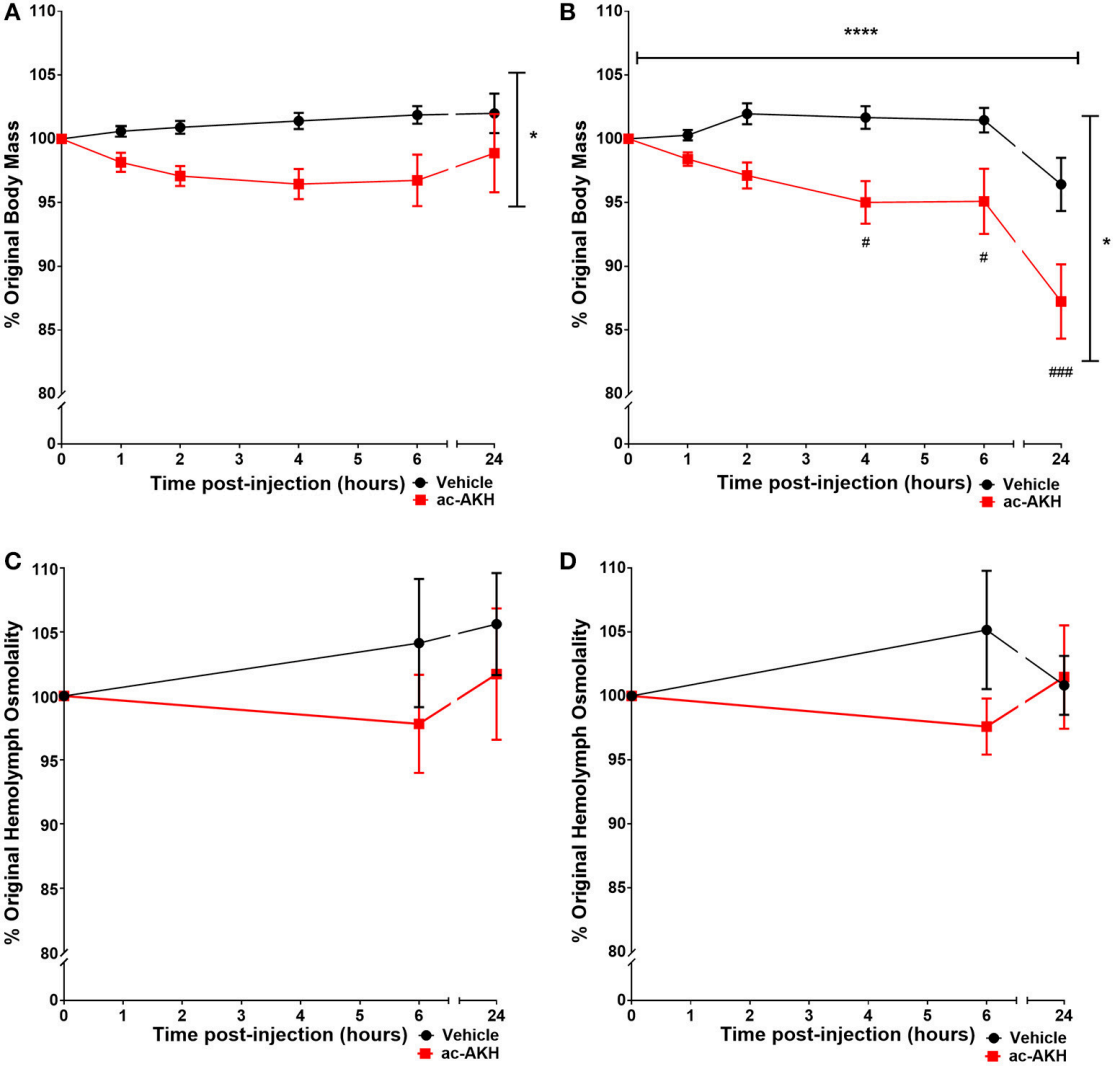
Percent change in body mass **(A, B)** and hemolymph osmolality **(C, D)** in response to a single injection of ASW vehicle or 10 μg ac-AKH in *A. californica* kept in a normosaline **(A, C)** or a hyposaline **(B, D)** condition. Each data point represents mean ± SEM, *n* = 6-7. Vertical and horizontal bars indicate a significant effect of treatment or time, respectively, measured by a two-way ANOVA (*denotes *p* < 0.05; **** denotes *p* < 0.0001). ac-AKH injection significantly reduced percent body mass at 4, 6, and 24 h in the hyposaline condition (# denotes *p* < 0.05, ### denotes *p* < 0.001). There was no significant effect of ac-AKH injection on hemolymph osmolality in either condition.

To examine if ac-AKH induced similar effects under different salinity conditions, animals kept under a normosaline or hyposaline condition were given a single injection of ASW vehicle or ac-AKH, and their body mass measured at regular intervals. For animals kept under a normosaline condition, the initial mean body mass was 140.15 ± 4.20 g, and the initial mean hemolymph osmolality was 903.77 ± 22.04 mOsm/L. Two-way ANOVA revealed a significant effect of treatment (ac-AKH injection) [*F*_(1, 65)_ = 5.482, *p* = 0.0391], but not time [*F*_(5, 65)_ = 0.666, *p* = 0.6508] or treatment x time interaction [*F*_(5.65)_ = 1.699, *p* = 0.1502], on body mass (Figure 1A). Bonferroni's *post-hoc* test revealed no significant differences between the treatment groups at any time point. For animals kept under a hyposaline condition, the initial mean body mass was 163.62 ± 8.29 g, and the initial mean hemolymph osmolality was 689.62 ± 10.11 mOsm/L. Two-way ANOVA revealed a significant effect of time [*F*_(5, 65)_ = 14.76, *p* < 0.0001], treatment [*F*_(1, 65)_ = 8.737, *p* = 0.0131], and treatment x time interaction [*F*_(5, 65)_ = 2.385, *p* = 0.0475] on body mass was observed (Figure 1B). Bonferroni's *post-hoc* test revealed that a single ac-AKH injection significantly reduced body mass at 4, 6, and 24 h under a hyposaline condition (Figure 1B). No significant differences in hemolymph osmolality were observed (Figures 1C,D). Additionally, no significant effects of ac-AKH on specific osmolyte concentrations were observed under the normosaline (Table 1) or hyposaline (Table 2) condition.

**Table 1:** Effects of ac-AKH injection on hemolymph osmolyte concentrations under a normosaline condition.

**Osmolyte**	**ASW 0 h**	**AKH 0 h**	**ASW 6 h**	**AKH 6 h**	***p*-value**
Na^+^ (mEq/L)	472.9 ± 4.557	464 ± 4.888	468.2 ± 8.032	467.5 ± 12.21	Not significant
K^+^ (mEq/L)	11.48 ± 0.080	11.22 ± 0.1158	11.38 ± 0.2188	11.4 ± 0.6996	Not significant
Cl^−^ (mEq/L)	536.6 ± 5.19	525.8 ± 5.31	531 ± 9.227	531.1 ± 14.76	Not significant
Ca^2+^ (g/dL)	45.33 ± 0.2611	44.72 ± 0.2893	45.15 ± 1.868	45.13 ± 1.237	Not significant
Glucose (mg/dL)	2.8 ± 0.8	2.2 ± 0.3742	2.8 ± 0.6633	2.8 ± 0.4899	Not significant
Proteins (mg/dL)	62.4 ± 6.265	70 ± 11.06	60.2 ± 13.9	71.8 ± 8.423	Not significant
Triglycerides (mg/dL)	Not detected	Not detected	Not detected	Not detected	N/A

*Concentrations are reported as the mean ± SEM, n = 5. No significant differences were observed*.

**Table 2:** Effects of ac-AKH injection on hemolymph osmolyte concentrations under a hyposaline condition.

**Osmolyte**	**ASW 0 h**	**AKH 0 h**	**ASW 6 h**	**AKH 6 h**	***p*-value**
Na^+^ (mEq/L)	364.7 ± 23.57	352.7 ± 2.221	320.5 ± 7.243	344.4 ± 9.555	Not significant
K^+^ (mEq/L)	9.028 ± 0.6398	8.562 ± 0.06492	7.988 ± 0.171	8.356 ± 0.2181	Not significant
Cl^−^ (mEq/L)	412.5 ± 26.34	398 ± 2.525	361.8 ± 8.08	389 ± 10.43	Not significant
Ca^2+^ (g/dL)	35.14 ± 2.31	30.68 ± 0.7106	34.34 ± 0.3811	32.49 ± 1.296	Not significant
Glucose (mg/dL)	3.2 ± 0.4899	3.6 ± 0.2449	2.6 ± 0.4	3.4 ± 0.4	Not significant
Proteins (mg/dL)	85.2 ± 14.71	83 ± 2.646	69.4 ± 10.47	69 ± 10.63	Not significant
Triglycerides (mg/dL)	not detected	not detected	not detected	not detected	N/A

*Concentrations are reported as the mean ± SEM, n = 4–5. No significant differences were observed*.

### Experiment 2: the effects of ac-AKH on hemolymph quantity

**Figure 2:**
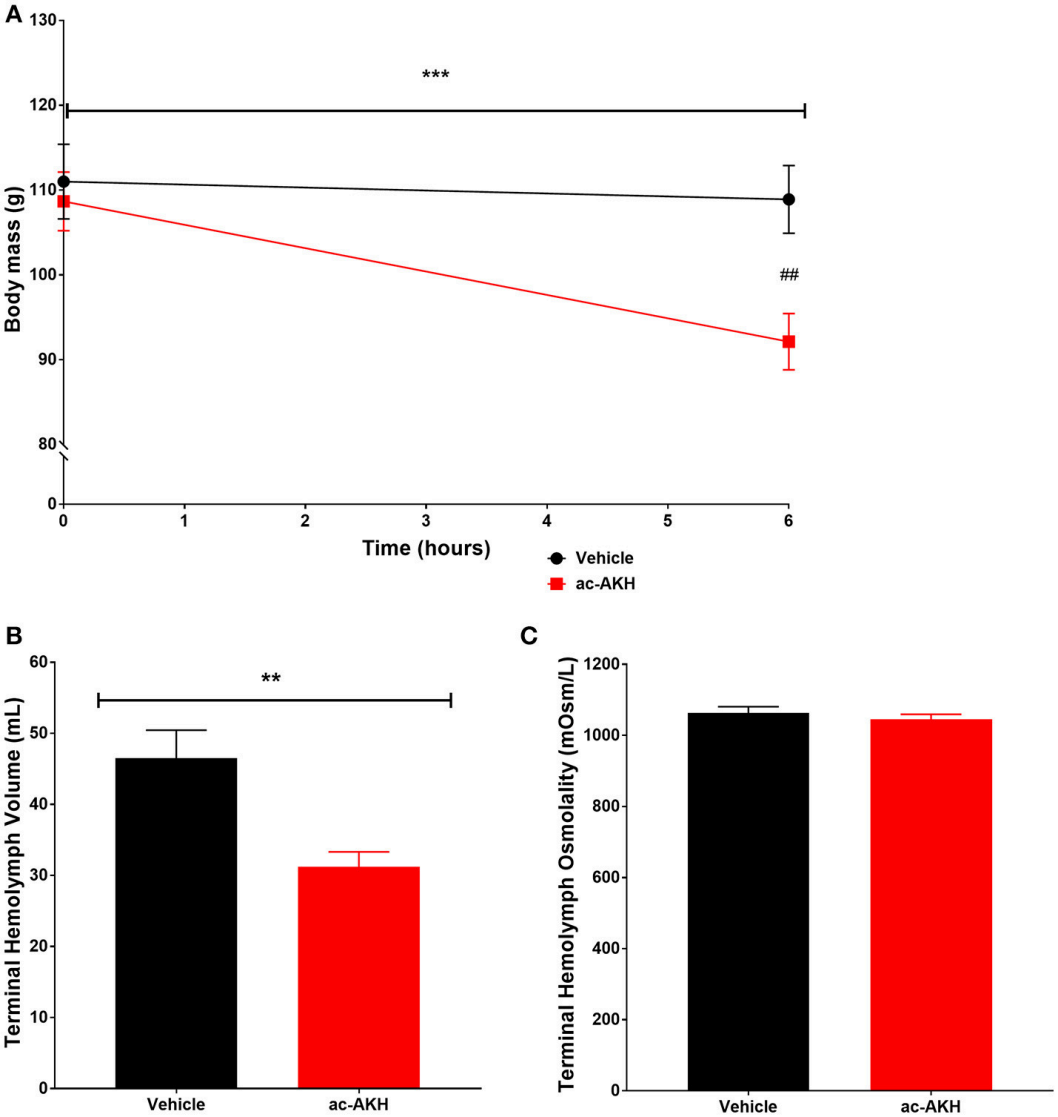
Change in absolute body mass **(A)**, terminal hemolymph volume **(B)**, and terminal hemolymph osmolality **(C)** in response to a single injection of ASW vehicle or 10 μg ac-AKH 6 h prior to sacrifice. Each data point and bar for **(A,B)** represents mean ± SEM, *n* = 7, and each bar for **(C)** represents mean ± SEM, *n* = 6. For **(A)**, a two-way ANOVA revealed a significant effect of time, but not treatment, on body mass (*** denotes *p* < 0.001). A significant difference in body mass between groups at 6 h was observed (## denotes *p* < 0.01). For **(B)**, Student's *t*-test measured a significant difference in terminal hemolymph volume between groups (** denotes *p* < 0.01). For **(C)**, Student's *t*-test measured no significant difference in terminal hemolymph osmolality.

We hypothesized that the primary mechanism of ac-AKH induced acute weight loss in Experiment 1 was reduction of hemolymph volume. We tested this hypothesis by measuring the terminal hemolymph volume at 6 h after an ASW vehicle or ac-AKH injection. Two-way ANOVA revealed a significant effect of time [*F*_(1, 14)_ = 26.92, *p* = 0.0002] and treatment x time interaction [*F*_(1, 14)_ = 16.11, *p* = 0.0017] but not treatment [*F*_(1, 14)_ = 3.511, *p* = 0.0855] on absolute body mass of animals injected with a single 10 μg dose of ac-AKH (Figure 2). Bonferroni's *post-hoc* test revealed that treatment significantly reduced absolute mass at 6 h post-injection (Figure 2A). Student's *t*-test revealed a significant reduction in absolute terminal hemolymph volume (Figure 2B) but no difference in absolute terminal hemolymph osmolality (Figure 2C) in animals injected with ac-AKH.

### Experiment 3: localization of ac-AKH in peripheral and central tissues

**Figure 3:**
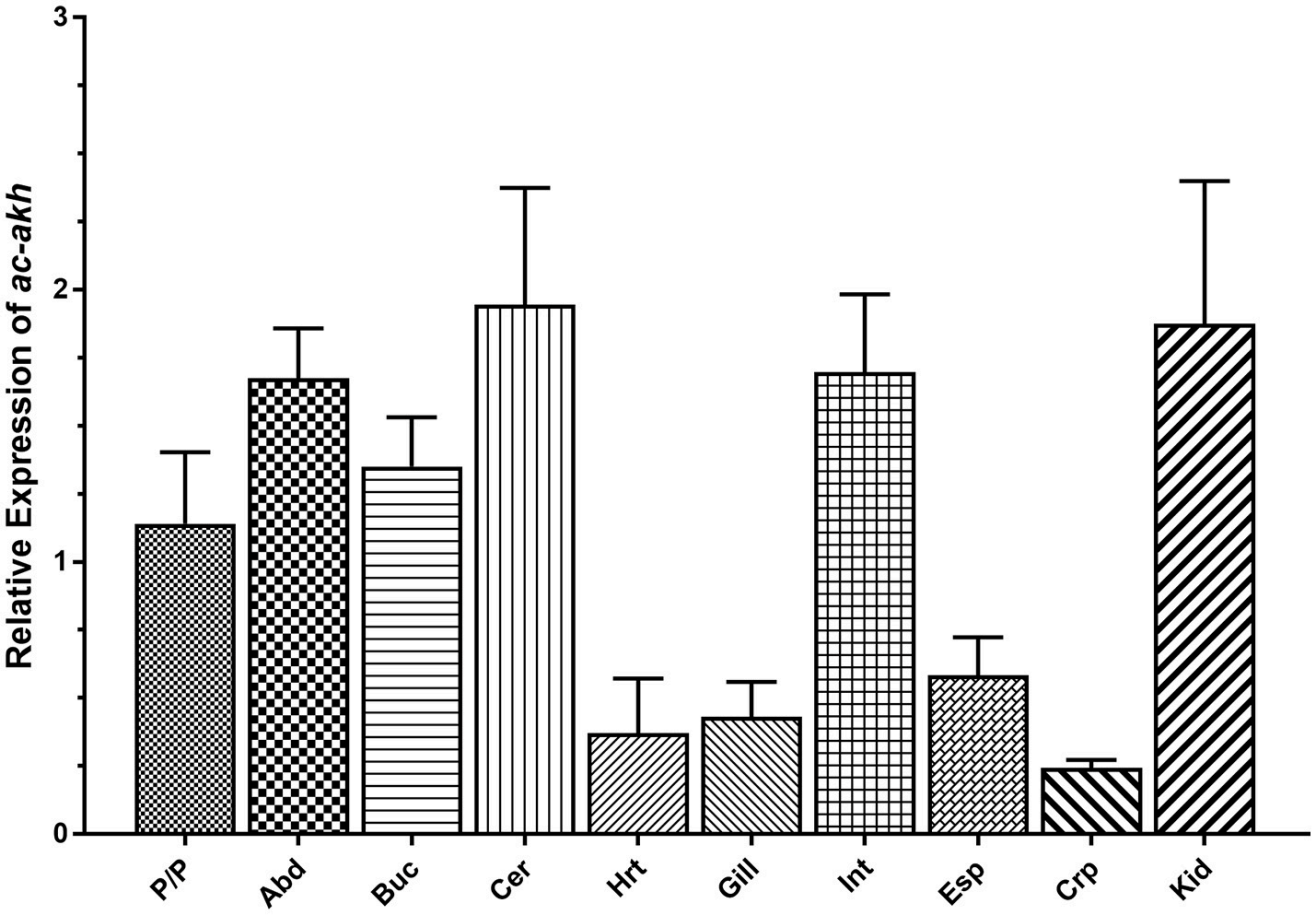
Relative expression of *ac-akh* in the pedal and pleural ganglia (P/P), the abdominal ganglion (Abd), the buccal ganglion (Buc), the cerebral ganglion (Cer), the heart (Hrt), the gill (Gill), the intestine (Int), the esophagus (Esp), the crop (Crp), and the kidney (Kid). Each bar represents mean ± SEM, *n* = 5. Statistical analysis was not performed for this study.

qPCR was performed to examine the abundance and distribution of ac-AKH transcript in the CNS and peripheral tissues. Abundant *ac-akh* was observed in all central ganglia including the abdominal, buccal, cerebral, and pedal/pleural ganglia, and in two peripheral organs associated with osmoregulation, the intestine and the kidney. Low levels of *ac-akh* expression were detected in the remaining tissues examined (Figure 3).

### Experiment 4: alteration of *ac-AKH* expression during normosaline to hyposaline transfer

Since ac-AKH may be involved in fluid homeostasis, we tested if its expression could be altered by a change in salinity. Animals transferred from a normosaline to hyposaline condition showed a significant inhibition in *ac-akh* transcript exclusively in the abdominal ganglion after 6 h (Table 3). This change was accompanied by an increase in percent original body mass (99.99 ± 0.341% in retained vs. 118.13 ± 0.764% in transferred; *p* < 0.0001) and a decrease in hemolymph osmolality in the transferred animals (970.13 ± 16.2 mOsm in retained vs. 851.63 ± 33.28 mOsm in transferred; *p* < 0.01).

**Table 3:** Effects of hyposaline transfer on the expression of *ac-akh*.

**Tissue**	**2^−ΔΔCT^ Retained**	**2^−ΔΔCT^ Transferred**	***p*-value**
Abdominal Ganglion	1.022 ± 0.1014	0.564 ± 0.08047	0.0083
Buccal Ganglion	1.26 ± 0.3214	2.802 ± 1.443	Not significant
Cerebral Ganglion	1.172 ± 0.2638	0.808 ± 0.1509	Not significant
Pedal/Pleural Ganglion	1.02 ± 0.1206	0.764 ± 0.1071	Not significant
Heart	2.168 ± 1.138	1.678 ± 0.655	Not significant
Intestine	3.233 ± 2.444	1.18 ± 0.3068	Not significant
Kidney	1.408 ± 0.4525	1.983 ± 0.5262	Not significant

*Each value represents mean ± SEM, n = 4–5*.

*Student's t-test showed a significant reduction in ac-akh transcript in the abdominal ganglion only*.

### Experiment 5: the effects of ac-AKH on hyposaline transfer

**Figure 4:**
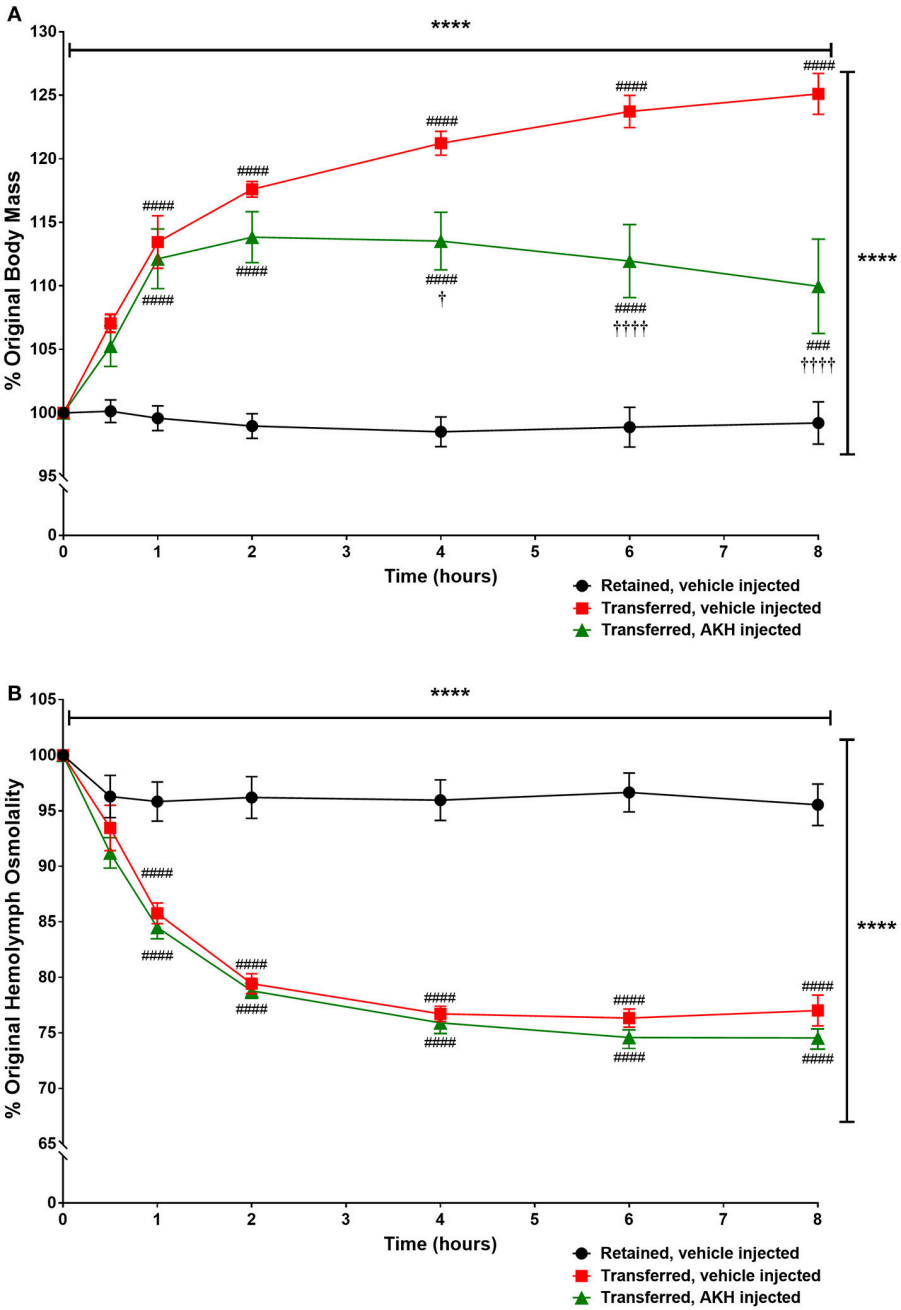
Percent change in **(A)** body mass or **(B)** hemolymph osmolality in *A. californica* either retained in a normosaline condition and injected with ASW vehicle, transferred to a hyposaline condition and injected with ASW, or transferred to a hyposaline condition and injected with 10 μg ac-AKH. Each data point represents mean ± SEM, *n* = 5. Vertical and horizontal bars indicate a significant effect of treatment and time, respectively, measured by a two-way ANOVA (**** denotes *p* < 0.0001). For **(A)**, significant differences were observed at several time points between the retained group and both transferred groups (### denotes *p* < 0.001, #### denotes *p* < 0.0001), and between the ASW-injected transfer group and the ac-AKH-injected transfer group († denotes *p* < 0.05, †††† denotes *p* < 0.0001). For **(B)**, significant differences were observed at several time points between the retained group and the transferred groups (#### denotes *p* < 0.0001), but not between the two transferred groups.

Since transfer to a hyposaline condition increased body mass in *A. californica*, we examined if ac-AKH could partially reverse the hyposalinity-induced weight gain. Two-way ANOVA revealed a significant effect of time [*F*_(6, 90)_ = 40.8, *p* < 0.0001], treatment (from normo- to hypo-saline condition) [*F*_(2, 90)_ = 42.67, *p* < 0.0001], and treatment x time interaction [*F*_(6, 90)_ = 18.6, *p* < 0.0001] on body mass (Figure 4). Bonferroni's *post-hoc* test revealed that the transfer to a hyposaline condition significantly increased the body mass, but this increase was reversed by ac-AKH injection at 4, 6, and 8 h (Figure 4). Similarly, a time [*F*_(6, 90)_ = 257.7, *p* < 0.0001], treatment [*F*_(2, 90)_ = 52.66, *p* < 0.0001], and treatment x time interaction [*F*_(6, 90)_ = 45.18, *p* < 0.0001] had a significant effect on hemolymph osmolality. Bonferroni's *post-hoc* test revealed that although the transfer to hyposaline condition significantly decreased hemolymph osmolality, in contrast to its ability to reverse the body mass gain, ac-AKH injection did not affect the decline in hemolymph osmolality (Figure 4).

## Discussion

For the first time, we report a potent effect of AKH treatment on the volume regulation in a marine gastropod mollusk, *A. californica*. We find that ac-AKH induces an acute volume loss while maintaining a hemolymph osmolality isotonic to the environment. Further, the hyposaline-induced downregulation of *ac-akh* in the abdominal ganglion, a ganglion responsible for key physiological functions such as reproduction, osmoregulation, circulation, and respiration (27), suggests *ac-akh* expression responds to the environmental salinity and the animal's internal fluid volume and composition. Collectively, our results demonstrate a novel physiological role of an AKH in the lophotrochozoan lineage and emphasize the functional diversity of the AKH family.

We do not believe the volume regulating effect of ac-AKH is pharmacological for two reasons. First, animals previously injected with a higher dose of ac-AKH (15 μg/animal) exhibited no signs of desensitization and continued to respond to repeated injections for up to 20 days. Second, injection with a higher dose (30 μg/animal) of a related peptide [*A. californica* GnRH; Supplementary Table 1, (28–37)] did not induce weight loss, suggesting the effects of injected ac-AKH are not a general pharmacological response to an injected neuropeptide, but rather something specific and unique to ac-AKH (11).

The effect of ac-AKH on body mass reduction is stronger and longer-lasting under a hyposaline condition compared to a normosaline condition (Figures 1A,B). This suggests part of the physiological response to the hyposaline condition is increased responsiveness to ac-AKH. The mechanism for this sensitization is unclear, but may be related to a key osmoregulatory neuron in the abdominal ganglion, R15. R15 is a spontaneously bursting neuron that releases a presumptive hormone, R15α1, capable of increasing water retention (14–16, 38), an effect opposite to ac-AKH. Since the activity of R15 is inhibited under a hyposaline condition (17, 20), one possibility is that ac-AKH may synergize with the hyposaline condition to further inhibit R15, thereby leading to a greater volume loss.

We have confirmed that the ac-AKH-induced volume loss was due, in part, to the loss of hemolymph (Figure 2B). Previous and current studies have shown that a volume change induced by a hyposaline transfer was associated with a decrease in hemolymph osmolality for up to 6 h in *A. californica* and 24 h in *A. brasiliana* (16, 20), indicating volume disruption is typically coupled with a short-term hemolymph osmolality change. However, the present study shows that this is not the case for volume loss induced by injected ac-AKH. The mechanism by which ac-AKH induces volume loss while maintaining, in lockstep, the hemolymph osmolality and osmolyte concentrations identical to those of control animals is at present unclear. We posit that ac-AKH induces volume loss without disrupting hemolymph osmolality by promoting the excretion of fluid isotonic to hemolymph. The mechanism underlying this type of fluid loss remains to be explored.

The distribution of *ac-akh* (Figure 3) further supports the hypothesis that ac-AKH is involved in volume regulation. We have previously localized the ac-AKH peptide in the abdominal, cerebral, and pleural ganglia (11), thus the expression of *ac-akh* in these ganglia is expected. We have also shown elevated expression of *ac-akh* in the intestine, an osmoregulatory tissue (39–41), a result consistent with the previous observation that ac-AKH-induced defecation in *A. californica*, possibly by altering gut motility (11). Finally, we find higher levels of expression of *ac-akh* in the kidney, a tissue associated with osmoregulation and volume regulation in vertebrates (42) and *A. californica* (43). However, the expression of *ac-akh* in the gill, a tissue associated with osmoregulation in teleost fishes (44) and *A. californica* (27), is very low (Figure 3). Overall, the expression pattern of *ac-akh* in the periphery supports the role of ac-AKH as a volume regulator.

We have previously found no evidence of ac-AKH immunoreactive cells in the periphery (11), suggesting peripheral tissues do not have the capability of de novo ac-AKH synthesis. We therefore hypothesize that the *ac-akh* transcript presently detected in the peripheral organs (Figure 2) represents mRNA transported to and stored in the axon terminals of the target organs and locally translated at levels below detection (45). This suggests ac-AKH may act as a neurotransmitter/neuromodulator, a role previously shown in crustaceans for a member of the AKH family, RPCH (10, 46) to influence its targets. Supporting this notion, ac-AKH immunoreactivity is not present in R15 but found in a few smaller unidentified neurons and abundant fibers impinging on multiple abdominal ganglion neurons and in the neuropil region of the CNS (11). Since the neuropil region concentrates efferent fibers, it is possible that ac-AKH acts as a neurotransmitter/neuromodulator to influence the activity of R15 and peripheral targets to promote fluid loss. Alternatively, a change in the endogenous burst activity of R15 may influence the transcription of *ac-akh*, leading to the altered control of peripheral targets.

We additionally show that a hyposaline transfer induces a downregulation of *ac-akh* in the abdominal ganglion (Table 3). This would be counterintuitive for an adaptive osmoregulatory response to a hyposaline challenge since ac-AKH promotes fluid loss, but would be consistent with a volume gain during a hyposaline transfer if the animal behaves as an osmoconformer. Although Skinner and Peretz (1989) showed *A. californica* could act as an osmoregulator by achieving a full recovery of hemolymph osmolality and body mass 24 h after being transferred to 90% oceanic salinity, our current data suggest that *A. californica* become an osmoconformer in a more dilute environment of 80% oceanic salinity, possibly as an adaptive strategy to conserve energy. This shift between osmoregulation and osmoconformation is also observed in another intertidal *Aplysia* species, *A. juliana*, which behave as an osmoregulator under a slightly hyposaline environment but an osmoconformer under a more dilute environment (43). In this sense, ac-AKH may not be a peptide that maintains homeostasis by promoting osmoregulation, but rather one that aids in the transition to osmoconformation. *A. californica* is an intertidal species that needs to withstand substantial salinity changes. For example, *A. californica* stranded in a small tide pool experience more extreme fluctuations in environmental salinity due to rainfall or evaporation (13). Changes in ac-AKH levels may allow *A. californica* to gain or lose fluid so its internal osmolality conforms with the environmental osmolality. Ultimately, our data suggest that ac-AKH likely has an important role in volume regulation but not necessarily osmoregulation.

We show that the administration of ac-AKH is capable of partially reversing the body mass gain following a hyposaline transfer (Figure 4). Since *ac-akh* is downregulated by a hyposaline challenge, we posit that ac-AKH has a physiological role in promoting a constitutive fluid loss, and the weight gain resulting from a hyposaline challenge is at least in part a consequence of decreased ac-AKH. In other words, reduced ac-AKH is needed for the animal to gain volume. This reversal of the body mass gain induced by ac-AKH is, again, not coupled to osmoregulation. In vertebrates, hormone-induced volume regulation and osmoregulation are closely linked (47, 48), but the current study shows that ac-AKH affects hemolymph volume without significantly altering hemolymph osmolality or specific osmolytes.

The discovery of AKH members in the lophotrochozoan lineage is relatively new (2), and they are currently neuropeptides in search of a function. Our results show, for the first time, that a lophotrochozoan AKH acts as a volume regulator without altering the hemolymph osmolality. We also show that this effect of ac-AKH is accentuated under a hyposaline condition, the expression of *ac-AKH* is inhibited under a hyposaline condition, and the administration of ac-AKH is capable of partially reversing the increase in body mass, but not a decline in hemolymph osmolality, induced by a hyposaline transfer. Collectively, these data underscore a novel mechanism of volume regulation induced by ac-AKH. Further, they suggest a need to explore a role of AKH in volume and osmo-regulation among additional members of the lophotrochozoan lineage.

## Ethics statement

As *Aplysia californica* are invertebrate animals, the approval of IACUC was not required. However, every effort was made to adhere to humane standards of treatment for care and use of these animals.

## Author contributions

AM and P-ST conceived and designed the experiments. AM performed the experiments and statistical analysis. AM and P-ST wrote and revised the manuscript.

### Conflict of interest statement

The authors declare that the research was conducted in the absence of any commercial or financial relationships that could be construed as a potential conflict of interest.
