# Supplementary material for: An Adipokinetic Hormone Acts as a Volume Regulator in the Intertidal Gastropod Mollusk, Aplysia californica
Source: Front Endocrinol (Lausanne). 2018 Aug 24;9:493. doi: 10.3389/fendo.2018.00493 (PMC6117392; doi:10.3389/fendo.2018.00493)
Supplement: Supplementary file 1 [file Table_1.DOCX]

Supplementary Material

An adipokinetic hormone acts as a volume regulator in the intertidal gastropod mollusk, *Aplysia californica*

Anthony W. Martillotti and Pei-San Tsai^*^

***Correspondence:**Pei-San Tsai
pei-san.tsai@colorado.edu

# Supplementary Tables

Table 1. Peptide sequences of selected AKHs and related peptides. Citations are found in the References section.

| **Peptide** | **Amino Acid Sequence** | **Citation** |
| --- | --- | --- |
| *Aplysia californica* AKH | pGlu-Ile-His-Phe-Ser-Pro-Asp-Trp-Gly-Thr-NH_2_ | Johnson *et al*., 2014 |
| Owl limpet (*Lottia gigantea*) AKH | pGlu-Ile-His-Phe-Ser-Pro-Thr-Trp-Gly-Ser-NH_2_ | Hauser and Grimmelikhuijzen, 2014 |
| Locust (*Locusta migratoria*) AKH-I | pGlu-Leu-Asn-Phe-Thr-Pro-Asn-Trp-Gly-Thr-NH_2_ | Stone *et al*., 1976 |
| Cockroach (*Periplaneta americana*) AKH | pGlu-Val-Asn-Phe-Ser-Pro-Asn-Trp-NH_2_ | Witten *et al*., 1984 Scarborough *et al*., 1984 |
| *Rhodnius prolixus* AKH | pGlu-Leu-Thr-Phe-Ser-Thr-Asp-Trp-NH_2_ | Marco *et al*., 2013 |
| Crustacean AKH/RPCH | pGlu-Leu-Asn-Phe-Ser-Pro-Gly-Trp-NH_2_ | Fernlund and Josefsson, 1972 |
| *Anopheles gambiae* ACP | pGlu-Val-Thr-Phe-Ser-Arg-Asp-Trp-Asn-Ala-NH_2_ | Hansen *et al*., 2010 |
| [His^7^]-Corazonin | pGlu‐Thr‐Phe‐Gln‐Tyr‐Ser‐Arg‐Gly‐Trp‐Thr‐Asn-NH_2_ | Veenstra, 1989 |
| *Aplysia californica* GnRH | pGlu-Asn-Tyr-His-Phe-Ser-Asn-Gly-Trp-Tyr-Ala-NH_2_ | Tsai *et al.*, 2010 |
| Owl limpet (*Lottia gigantea*) GnRH | pGlu-His-Tyr-His-Phe-Ser-Asn-Gly-Trp-Lys-Ser-NH_2_ | Sun *et al.*, 2012 |
| Chicken (*Gallus gallus*) GnRH-II | pGlu-His-Trp-Ser-His-Gly-Trp-Tyr-Pro-Gly-NH_2_ | Miyamoto *et al*., 1984 |
| Salmon (*Oncorhynchus keta*) GnRH | pGlu-His-Trp-Ser-Tyr-Gly-Trp-Leu-Pro-Gly-NH_2_ | Sherwood *et al*., 1983 |
